# Potsdam data set of eye movement on natural scenes (DAEMONS)

**DOI:** 10.3389/fpsyg.2024.1389609

**Published:** 2024-05-10

**Authors:** Lisa Schwetlick, Matthias Kümmerer, Matthias Bethge, Ralf Engbert

**Affiliations:** ^1^Department of Experimental and Biological Psychology, University of Potsdam, Potsdam, Germany; ^2^Tübingen AI Center, University of Tübingen, Tübingen, Germany

**Keywords:** eye movement, fixations, scan path, modeling, machine learning, saliency, data set

## 1 Introduction

In recent years, model-based simulation of eye-movement behavior has become a more and more powerful scientific tool to understanding visual cognition (Kümmerer and Bethge, 2023). Predicting eye movements requires understanding low-level vision properties, high-level cognitive aspects and taking image content into account. For this reason, the task of eye movement prediction has received a lot of attention from diverse fields such as vision science, cognitive science, and computer vision, as well as applied fields such as foveated rendering, compression, robotics, design. As eye-movement models increase in complexity, the role of high-quality, openly available data sets becomes increasingly crucial in advancing the field. Corpora, i.e., general-purpose data sets, serve as the foundation upon which researchers can train, test and validate their models. A powerful method that is particularly popular in the field of computer vision is rigorous benchmarking on commonly accepted data sets. The fair and statistically rigorous comparison of models is crucial in order to ascertain the behavioral relevance of hypothesized effects and mechanisms, to understand the advantages and shortcomings of different approaches, and to rank models with respect to their success in explaining behavior.

The different research traditions contributing to the field of modeling eye movement each prioritize different ideas, methods, specific interests and goals. Nonetheless the cross-pollination between the fields may be an important asset to further our understanding of human vision. One important difference between the various modeling approaches concerns the data requirements. In order to achieve the best possible standard, different models require different properties from a data set. As an example, we can compare two computational models of scan path generation: the Deep Neural Network model DeepGaze III (Kümmerer et al., 2019), and the dynamical, biologically-inspired model SceneWalk (Engbert et al., 2015; Schwetlick et al., 2020, 2022). DeepGaze III requires large amounts of data with many different image examples in order to fit many thousands of model parameters. SceneWalk, by contrast, has only a handful of parameters and needs comparatively much less data to fit. It does, however, rely on long sequences (i.e., long presentation durations) in order to fit sequence effects accurately.

The MIT/Tuebingen Saliency Benchmark (Kümmerer et al., 2018) is the commonly agreed-upon benchmark for free viewing saliency prediction on static images since 2013, when it was established as the MIT Saliency Benchmark (Judd et al., 2012). This benchmark scores the performance of fixation density prediction models (often denoted as “saliency models,” even though the term saliency originally only referred to low level features) on two different data sets with a public training part and a hold-out test part, to avoid overfitting to the test set.

Recently, there has been an increased interest in modeling not just the spatial distribution of fixations but also entire fixation sequences (scan paths), introducing sequence effects and temporal effects. While in principle the same data set could be used for both static and dynamic fixation prediction, in practice the requirements can differ. Some existing data sets use very short presentation times, resulting in relatively short scan paths with little information on sequential effects. In other public data sets, sequence and temporal data was not published initially and was lost. The issue is compounded by the fact that, even when data is published, preprocessing details are not consistently reported. This lack of transparency raises concerns about data quality, hindering retrospective addressing of the issue.

In this study we address the above shortcomings of data sets and collect and openly publish a scene viewing corpus data set which is useful for many different modeling approaches, including machine learning applications, principled cognitive modeling, as well as experimental analysis. The data set is large, including 2,400 unique images and 250 participants, and we assure high data quality by using a state-of-the-art experimental eye tracker, meticulous data preprocessing, and a thorough and principled saccade detection procedure. All data processing is documented at a high level of detail and raw data is archived. The data set is purpose-built to be a good basis for a benchmark, by comprising an extensive training, validation and test set. Thanks to the large variety in images, annotations, and subjects, the data set is also suitable for experimental analyzes. The resulting data set can be used by both computer vision and experimental research traditions, and will enable us to combine and compare insights from research of the visual system with state of the art machine learning techniques. It will also be invaluable in establishing a benchmark for scan path modeling as well as adding to the benchmark for spatial saliency as part of the established MIT/Tuebingen Saliency Benchmark.

## 2 Stimulus material

**Figure 1:**
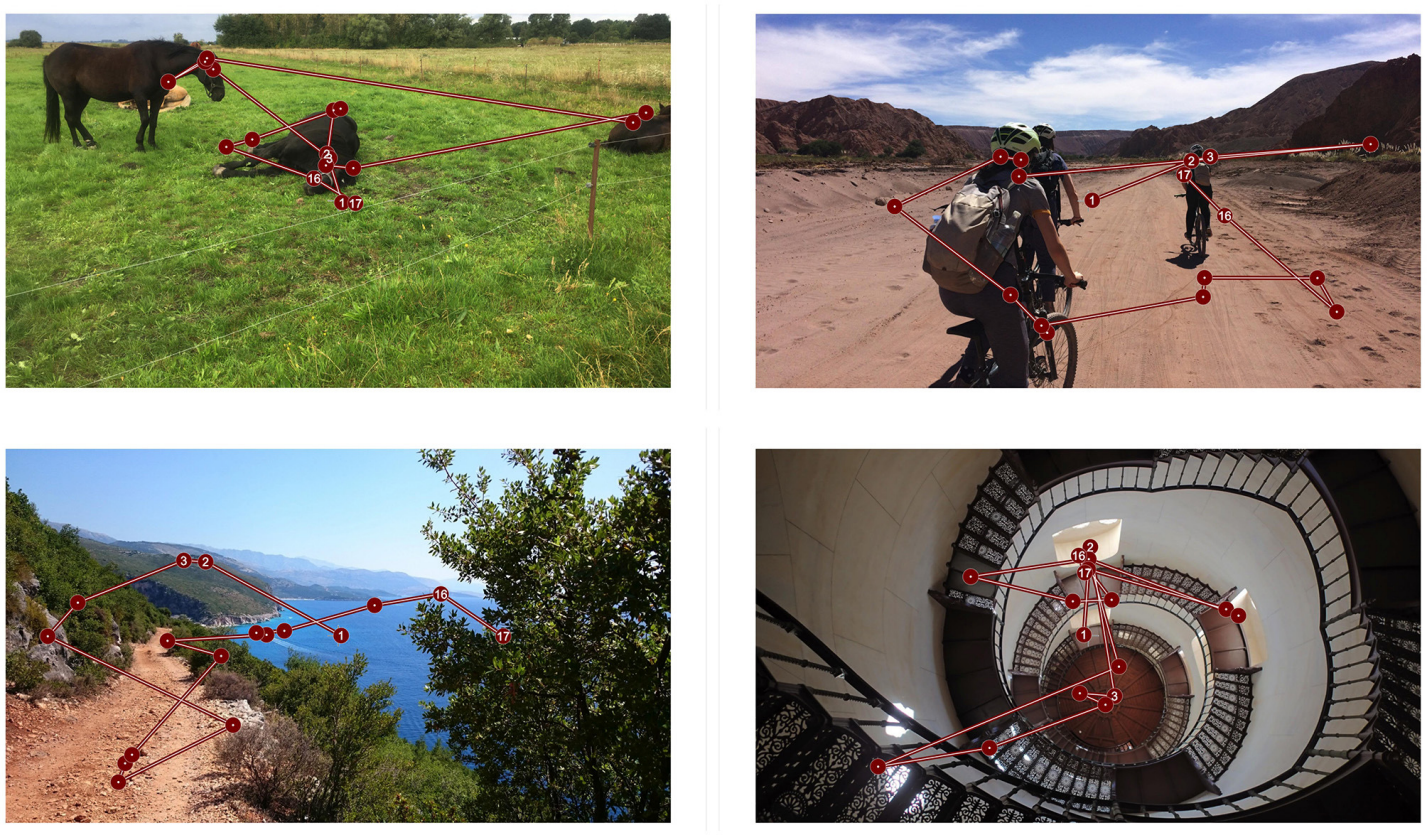
Example images with corresponding example scan paths. Each 8 s trial begins with a fixation on a central fixation marker. Circles represent fixations and the connecting lines represent saccades.

In this study, we collected eye tracking data on a set of 2,400 color photographs of natural scenes (see examples in Figure 1). The stimulus material was collected especially for this study. Photographs are in landscape format and have a resolution of 1,920 × 1,080, px. The rationale behind the choice of the format was to make use of high-resolution stimuli covering the full size of the ViewPixx Monitor used in our experiment. High resolution, large size stimuli are important to acquire natural eye movement behavior (von Wartburg et al., 2007; Otero-Millan et al., 2013).

In addition to the format considerations we also selected images to minimize central/photographers bias, unfocused regions (such as is frequent in portraits) and writing. We actively selected the data set to maximize variability both between and within images in terms of content and distribution of features or salient regions. One half of the images are freely available creative commons images taken from the platform *Flickr*.^1^ The other half are images taken by photographers, where we paid photographers to take pictures and release them into creative commons. Post-processing of the images involved adjusting the sizes of the images, typically by downsizing such that one edge matched our requirements and then trimming any overlap evenly from both sides, so as to maintain the center of the image. All 2,400 images are published on the Open Science Framework (see link in Section Data Availability).

The DAEMONS image data set, thus, is specifically designed to include high-resolution natural scenes with no artifacts (e.g., blurred faces to ensure privacy, as in the mapilary data set^2^) or large amounts of text (which would cause very idiosyncratic reading behavior), holding up to the standards of experimental cognitive science. While the commonly used, labeled machine learning data sets, which are not collected specifically for conducting behavioral measurements, are even larger (Lin et al., 2014; Russakovsky et al., 2015), the present high-resolution data set can be considered large enough to allow for commonly used machine learning methods to be applied. Information about the images is documented in the published repository.

### 2.1 Training, validation and test splits

The stimulus images were split into a training-, a validation-, and a test subset. The assignment of images to the three subsets was random with the only contraint that equally many Potsdam and Flickr images were in poth parts. The training subset comprises a large number of images (2,000), each seen by a smaller number of human observers (each image is seen by 10 participants). The validation and test subsets comprise a smaller number of images (200 validation and 200 test images) that were seen by larger numbers of observers (each image is seen by 50 participants).

### 2.2 Image annotations

We include image labels that were manually annotated by three people using a Javascript-based Tool.^3^ For each image we report a judgement for the following questions:

1. Is the scene inside or outside? [Inside, Outside].
2. Are any people visible? [Yes, No].
3. Is any text visible? [Yes, No].
4. Are any animals visible? [Yes, No].
5. How many salient objects are in this scene? [0 (meaning none or many), 1, 2, 3].

As the number of salient objects increases, each single one, by definition, becomes less salient. Individual salient objects strongly attract gaze behavior, for many or no salient objects the gaze is much less predictable. For this reason, we grouped the case of many salient objects together with the case of no salient object.

## 3 Eye tracking study

### 3.1 Participants

We invited 250 human observers to take part in the eye-tracking study. Participants received either course credit or monetary compensation for their involvement. Participants were between 16 and 64 years old; 184 participants were women, 65 men and one preferred not to say. Before the experiment participants' visual acuities were tested using the Freiburg Vision Test “FrACT,” Version 3.10.0 (Bach, 2006). The visual acuity test was conducted with glasses/contact lenses in order to ascertain normal or corrected-to-normal vision. In most cases participants who needed vision aids participated in the experiment wearing contact lenses. An exception was made for participants who's vision was fine at the viewing distance of the experiment (95 cm). Participants also filled out a 5-item questionnaire about their general wellbeing. The detailed participant information is provided together with the published data.

### 3.2 Experimental setup

The study was conducted in a quiet, dimly lit room. Participants were seated at a distance of 95 cm from a monitor, with their head securely held in place by a head-chin rest. An experimenter was present (but out of view) throughout the session for operating and calibrating the eye tracking system at regular intervals.

The stimuli were displayed on an ViewPixx Monitor with a resolution of 1,920 × 1,200 pixels and a refresh rate of 100–120 Hz. The eye-tracking experiment, including the presentation of stimuli and collection of responses, was controlled using MATLAB (version R2015b, MATLAB, 2015) in conjunction with the Psychophysics Toolbox (PTB-3; Brainard, 1997; Kleiner et al., 2007), which includes the Eyelink Toolbox (Cornelissen et al., 2002).

Eye tracking data were collected using an Eyelink 1000 eye tracker (1,000 Hz, binocularly). A 9-point calibration was repeated every 20 trials to ensure sufficient absolute gaze accuracy. The images were presented at the maximum trackable size for the Eyelink 1000 Desktop mount (i.e., 32° visual angle, 95 cm distance to the monitor). The illumination level of the tracker was set to 75%.

### 3.3 Design

Each participant saw 160 images from the stimulus data set described above for a duration of 8 s. Each trial began with a fixation marker in the center of a blank screen. The stimulus image appeared underneath the marker, before the marker disappeared after 175 ms. Participants were instructed to keep fixating the marker as long as it was present. This instruction was given in order to minimize the Central Fixation Bias, as shown by Rothkegel et al. (2017). Participants were instructed to blink as little as possible and to carefully explore the images.

From the test-, training and validation design (described in Section 2.1), it follows that each participant saw 80 training images and 80 test or validation images. Every 20 images, participants were given a recognition task to ensure attentive participation. The instruction was to view three images and then chose the unknown image between two previously seen images. Participants were rewarded for correct answers by an additional remuneration at the end of the study.

## 4 Data processing

The full eye tracking data processing pipeline is published as code alongside the data. First, timestamps denoting the beginning and end of a given trial were extracted to isolate the correct time windows of the eye tracking data. The raw data contain x and y coordinates of each eye at a recording rate of 1,000 Hz, as well as pupil size. Saccades were detected in the data stream using a velocity-based detection algorithm (Engbert and Kliegl, 2003), updated by Engbert and Mergenthaler (2006), using the relative threshold parameters λ = 4 and a minimum duration of five samples for saccades. As the detection algorithm is relatively sensitive under this parametrization, saccades that include an overshoot, and thereby a slow-down related to a change of direction, are frequently marked as two separate events by the algorithm. We corrected this effect by interpreting all fixations shorter than 25 ms as part of a saccade and adjusted the saccade parameters during postprocessing accordingly.

**Figure 2:**
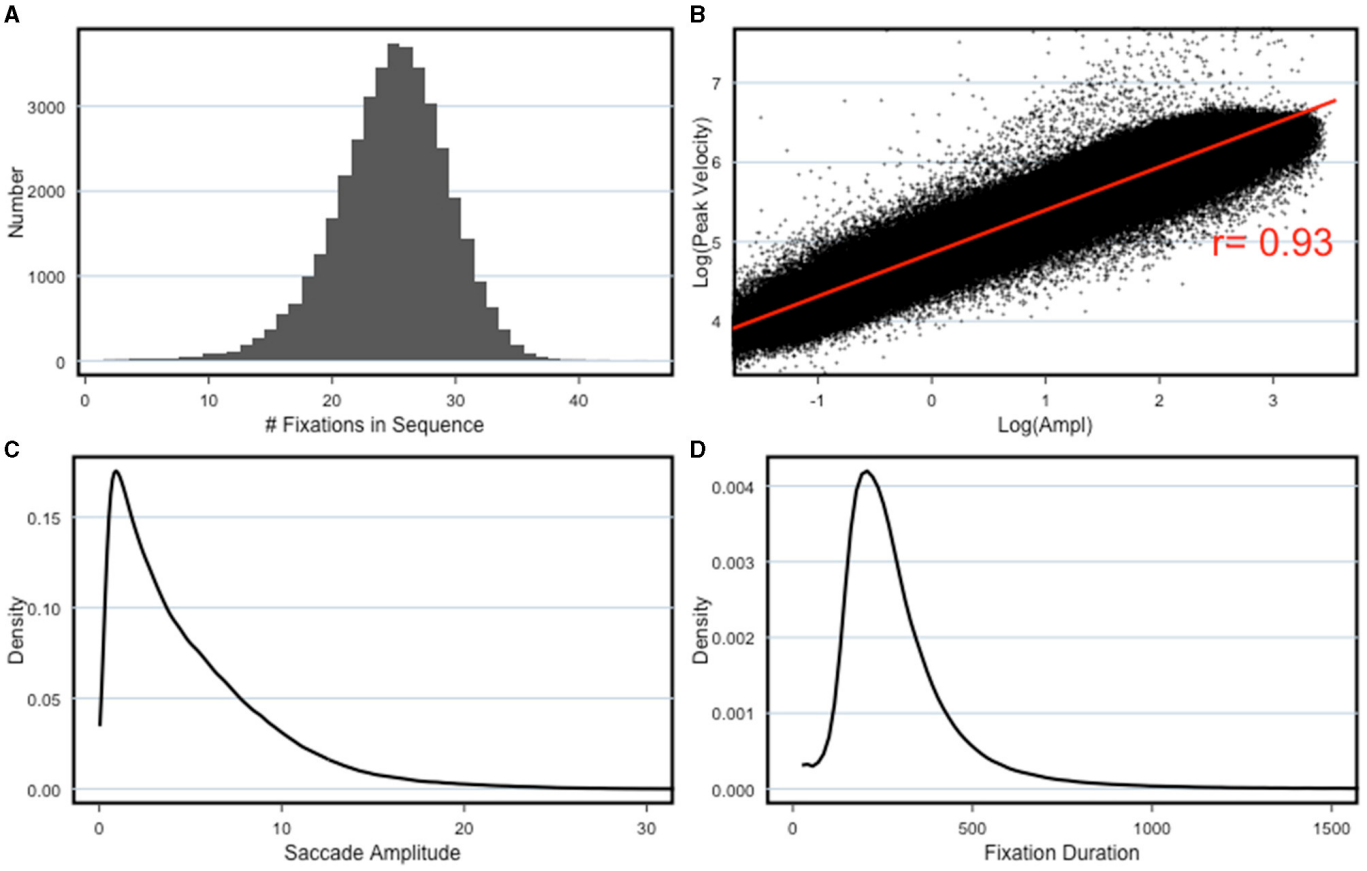
Saccade detection statistics. **(A)** Number of fixations per 8 s sequence. **(B)** Main sequence, showing the the Log Peak Velocity and Log Amplitude of the saccades are well-correlated. **(C)** Saccade amplitude distribution and **(D)** fixation duration distribution, which are both expected to be uni-modal, left-skewed distributions, peaking around 2 deg and 150 ms, respectively.

The parameters of the saccade detection pipeline were chosen manually by repeatedly investigating characteristic saccade and fixation statistics, as well as the performance of classifying individual events. Fixations and saccades have characteristic properties that are stable over viewers and experiments, which are well-documented in the literature. When detection performance is poor, the characteristic properties are less pronounced. An important example of a saccade statistic that is indicative of detection quality is the Main Sequence (Bahill et al., 1975). The log amplitude of saccades and their log peak velocity should be highly correlated. Figure 2B shows the Main Sequence for the presented data set. Further statistics include the number of fixations made (Figure 2A), the characteristic saccade amplitude distribution (Figure 2C) and fixation duration (Figure 2D), which should be uni-modal.

## 5 Database details

The published dataset, named DAEMONS (data set for eye movement on natural scenes) comprises two parts: the stimulus images and the eye tracking experiment.

The stimulus images are published at: https://osf.io/cn5yp/ as JPEG images (https://jpeg.org). There are two subsets, the images that were harvested from the plattform Flickr, and the Potsdam images, which were hand-collected by us. For each image we provide the photograph meta-data, as available. We also provide the image annotations in this folder.

Second, the eye tracking experiment is available at: https://osf.io/ewyg7/. Here we publish saccade tables that include columns for identifying information such as subject, trial and image number, as well as the beginning and end times and endpoint coordinates of saccades. A more detailed description of the columns may be found in the folder. We also publish supplementary anonymized information about each subject, such as age, gender, visual acuity, and their answers to the questionnaire. Raw data will be archived and documented for future reference. The computer code for the experiment and the data processing pipeline will be published separately.

## Data availability statement

The datasets presented in this study can be found in online repositories. The names of the repository/repositories and accession number(s) can be found at: https://osf.io/ewr5u/.

## Ethics statement

Ethical approval was not required for the studies involving humans because, ethical review and approval was not required for the study of eye movements on anonymized human participants in accordance with the local legislation and institutional requirements. The studies were conducted in accordance with the local legislation and institutional requirements. The participants provided their written informed consent to participate in this study.

## Author contributions

LS: Conceptualization, Data curation, Formal analysis, Investigation, Methodology, Project administration, Software, Supervision, Validation, Visualization, Writing – original draft, Writing – review & editing. MK: Conceptualization, Investigation, Validation, Writing – review & editing. MB: Conceptualization, Project administration, Supervision, Writing – review & editing. RE: Conceptualization, Funding acquisition, Methodology, Project administration, Resources, Supervision, Writing – review & editing.

## Footnotes

^1^ https://flickr.com

^2^ https://www.mapillary.com/dataset/vistas

^3^ github.com/lschwetlick/ImageAnnotationTool
